# Application of Implementation Science Principles in LMIC Settings – South Asian Exemplars

**DOI:** 10.1002/alz70858_098099

**Published:** 2025-12-24

**Authors:** Iracema Leroi

**Affiliations:** ^1^ Trinity College Institute of Neuroscience, Trinity College Dublin, Dublin, Ireland

## Abstract

**Background:**

Dementia research in low‐ and middle‐income countries (LMICs) faces numerous challenges, including limited resources, cultural barriers, and underdeveloped research infrastructure. Implementation science provides a structured approach to overcome these challenges by fostering co‐design, building research capacity, and promoting equitable global collaborations. The objective of this presentation is to outline three exemplars of applying implementation science principles for applied dementia research in a South Asian setting, highlighting: (1) patient and public involvement (PPI); (2) barriers to implementing culturally adapted non‐drug interventions for dementia; and (3) a framework for sustainable and equitable global partnerships in applied dementia research.

**Method:**

Three exemplar studies illustrate these objectives. The first study focused on the implementation of PPI, establishing groups at seven South Asian research sites. This initiative involved training researchers, adapting study protocols, and engaging the public using the Wellcome Trust's “Public Engagement Onion” model. The second study tested a culturally adapted hearing support intervention for individuals with dementia in Pakistan through a phased approach that included training, cultural adaptation, and home‐based intervention delivery by trained practitioners. The third study developed a framework for equitable global partnerships, emphasizing fairness, bidirectional learning, and stakeholder engagement to ensure inclusivity and sustainability in international collaborations.

**Results:**

The PPI initiative enhanced research capacity, ensured culturally relevant protocols, and raised public awareness of dementia. The hearing intervention was acceptable and feasible, though logistical barriers were identified and addressed to improve scalability. The equitable partnerships framework promoted fair practices, capacity building, and inclusivity, offering a model for sustainable collaborations.

**Conclusion:**

These studies demonstrate that co‐designed approaches, culturally adapted interventions, and equitable partnerships are essential to advancing dementia research in LMICs. By integrating these strategies, it is possible to create scalable, impactful solutions to address the growing burden of dementia in resource‐limited settings.

